# Supplementary material for: SARS-CoV-2 ORF7a potently inhibits the antiviral effect of the host factor SERINC5
Source: Nat Commun. 2022 May 26;13:2935. doi: 10.1038/s41467-022-30609-9 (PMC9135752; doi:10.1038/s41467-022-30609-9)
Supplement: Supplementary file 1 — Supplementary Information [file 41467_2022_30609_MOESM1_ESM.pdf]

Supplementary Information

SARS-CoV-2 ORF7a potently inhibits the antiviral effect of the host factor SERINC5

Uddhav Timilsina[1], Supawadee Umthong[1], Emily B Ivey[1], Brandon Waxman[1], Spyridon Stavrou[1*]

[1] Department of Microbiology and Immunology, Jacobs School of Medicine and Biomedical Sciences, University at Buffalo, NY, United States

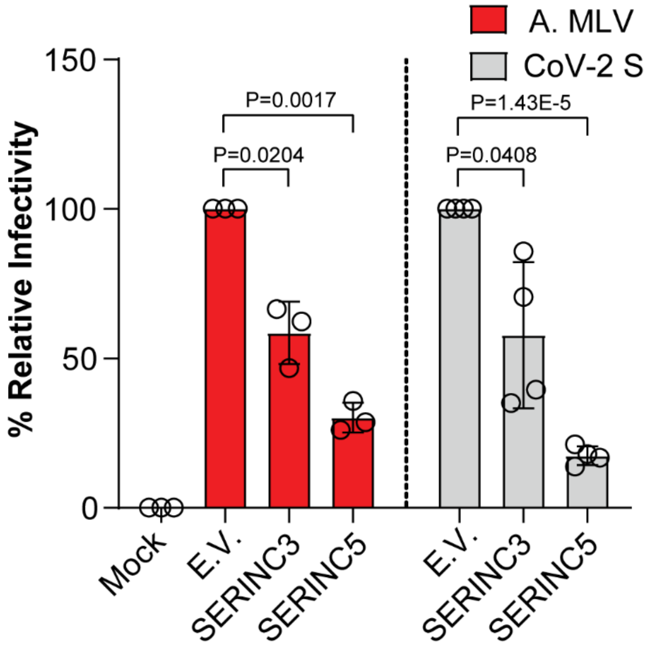

Supplementary Figure 1. **SERINC3 and SERINC5 restrict SARS-CoV-2 S pseudoviruses generated with a retroviral packaging system.** 293T-hACE2 cells were infected with either Amphotropic MLV envelope or SARS-CoV S pseudoviruses produced in the presence of SERINC3, SERINC5 or empty vector using a retroviral packaging plasmid. Luciferase levels were measured 48 hpi and normalized to MLV $p30^{CA}$ levels in the input pseudovirus. The percentage (%) of relative infectivity with respect to pseudovirus produced in the presence of empty vector is shown. All results are presented as mean ± SD from n = 3 (A. MLV) or n =4 (CoV-2 S) independent experiments. Statistical significance was determined by one-sample t-test (two-tailed). (empty vector, E.V.; SARS-CoV-2 Spike, CoV-2 S; Amphotropic MLV, A. MLV)

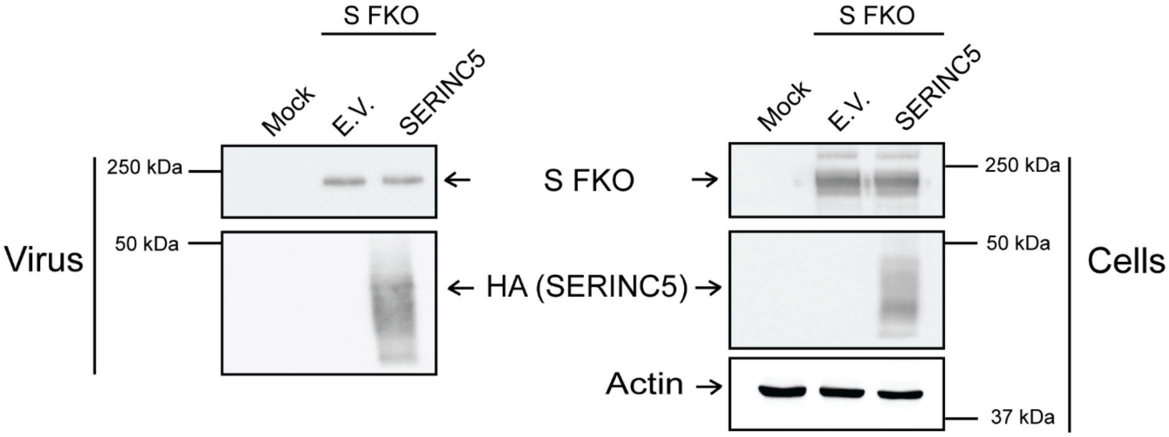

Supplementary Figure 2. **SERINC5 is incorporated in SARS-CoV-2 S FKO pseudoviruses.** 293T cells were co-transfected with HIV-1_{NL}ΔEnv-NanoLuc, SARS-CoV-2S-FKO and either SERINC5 or empty vector plasmids as indicated. Forty-eight hours post transfection, cells and released pseudovirus in the culture media were harvested and the indicated proteins were analyzed by immunoblotting. Representative immunoblotting results are shown for n = 3 independent experiments. Uncropped blots are in Source Data. (empty vector, E.V.; SARS-CoV-2 S-FKO, S FKO)

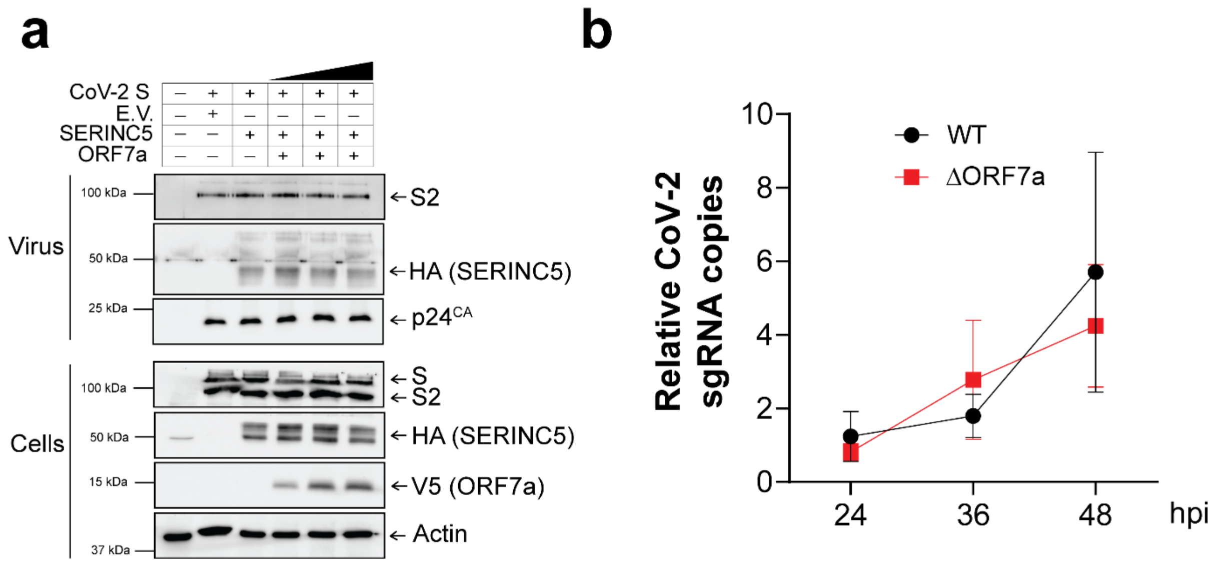

Supplementary Figure 3. **Immunoblot analyses of SARS-CoV-2 pseudoviruses produced in the presence of SERINC5 and SARS-CoV-2 ORF7a, and replication of SARS-CoV-2 WT and eGFP (ΔORF7a) viruses in 293T-hACE2 cells. a** 293T cells were co-transfected with HIV-1_{NL}ΔEnv-NanoLuc, SARS-CoV-2 Spike and either SERINC5, empty vector or different amounts of SARS-CoV-2 ORF7a. Forty-eight hours post transfection, cells and released pseudovirus in the culture media were harvested and the indicated proteins were analyzed by immunoblotting. Representative immunoblotting results are shown for n = 3 independent experiments. Uncropped blots are in Source Data. **b** SARS-CoV-2 WT and SARS-CoV-2 ΔORF7a viruses replicate similarly in 293T-hACE2 cells. 293T-hACE2 cells were infected with equal amounts of SARS-CoV-2 WT or ΔORF7a virus. At the indicated time points post infection, SARS-CoV-2 subgenomic (Spike) RNA copy number in the infected cells were determined by RT-qPCR and normalized to GAPDH. Results are shown as mean ± SD for n =3 independent experiments. (empty vector, E.V.; SARS-CoV-2 Spike, S; SARS-CoV-2 Spike S2 subunit, S2; SARS-CoV-2 ORF7a, ORF7a; subgenomic RNA, sgRNA; hours post infection, hpi)

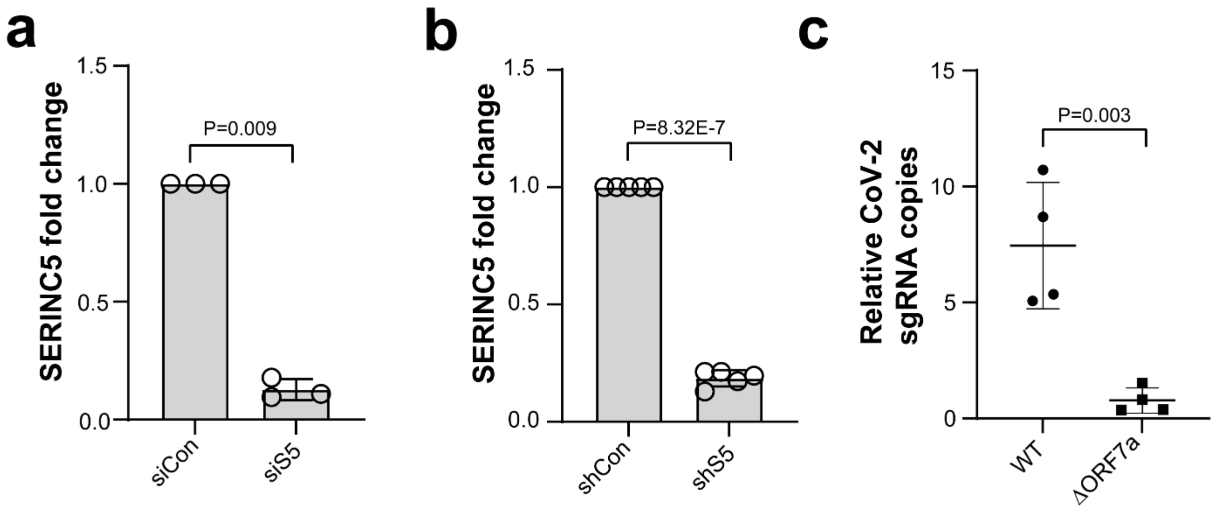

Supplementary Figure 4. **SERINC5-specific siRNA/shRNA knockdown verification in Calu-3 cells and replication of SARS-CoV-2 WT and eGFP (ΔORF7a) viruses in Calu-3 cells. a** Fold expression change of SERINC5 transcripts relative to negative control siRNA transfected, normalized to GAPDH in Calu-3 cells at 72 hours post transfection. Results are shown as mean ± SD for n = 3 independent experiments. **b** Fold expression change of SERINC5 transcripts in Calu-3 cells stably expressing SERINC5-shRNA relative to negative control shRNA transduced Calu-3 cells, normalized to GAPDH. Results are shown as mean ± SD for n = 5 independent experiments. St). **c** SARS-CoV-2 WT and SARS-CoV-2 ΔORF7a viruses replicate differently in Calu-3 cells at 24 hpi. Calu-3 cells were infected with equal amounts of SARS-CoV-2 WT or ΔORF7a virus. At 24 hpi, SARS-CoV-2 subgenomic (Spike) RNA copy number in the infected cells were determined by RT-qPCR and normalized to GAPDH. Results are shown as mean ± SD for n = 4 independent experiments. For (a and b) statistical significance was measured by one-sample t-test (two-tailed) and for (c) was determined by unpaired t-test (two-tailed). (negative control siRNA, siCon; SERINC5 siRNA, siS5; negative Control shRNA, shCon; SERINC5 shRNA, shS5, subgenomic RNA, sgRNA)

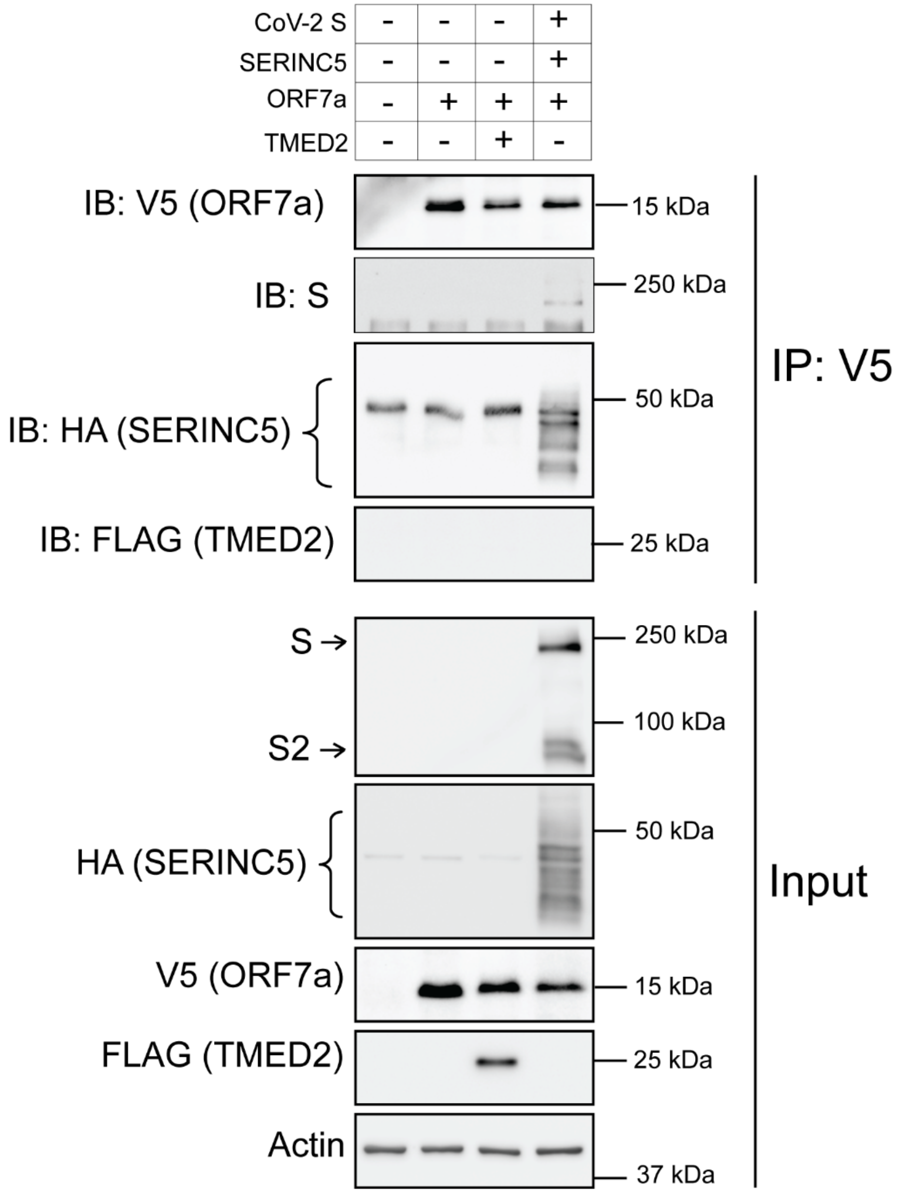

Supplementary Figure 5. **SARS-CoV-2 ORF7a doesn't interact with TMED2, an ERGIC resident protein.** 293T cells were cotransfected with SARS-CoV-2 S, SERINC5, SARS-CoV-2 ORF7a, TMED2 or empty vector as indicated. Cells were harvested 24 hours post transfection and lysates were immunoprecipitated with anti-V5 antibody followed by immunoblot analyses probing with anti-SARS-CoV-2 S, anti-HA (SERINC5), anti-V5 (SARS-CoV-2 ORF7a), anti-FLAG and anti-Actin antibodies. Representative immunoblotting results from n = 3 independent experiments are shown. Uncropped blots are in Source Data.

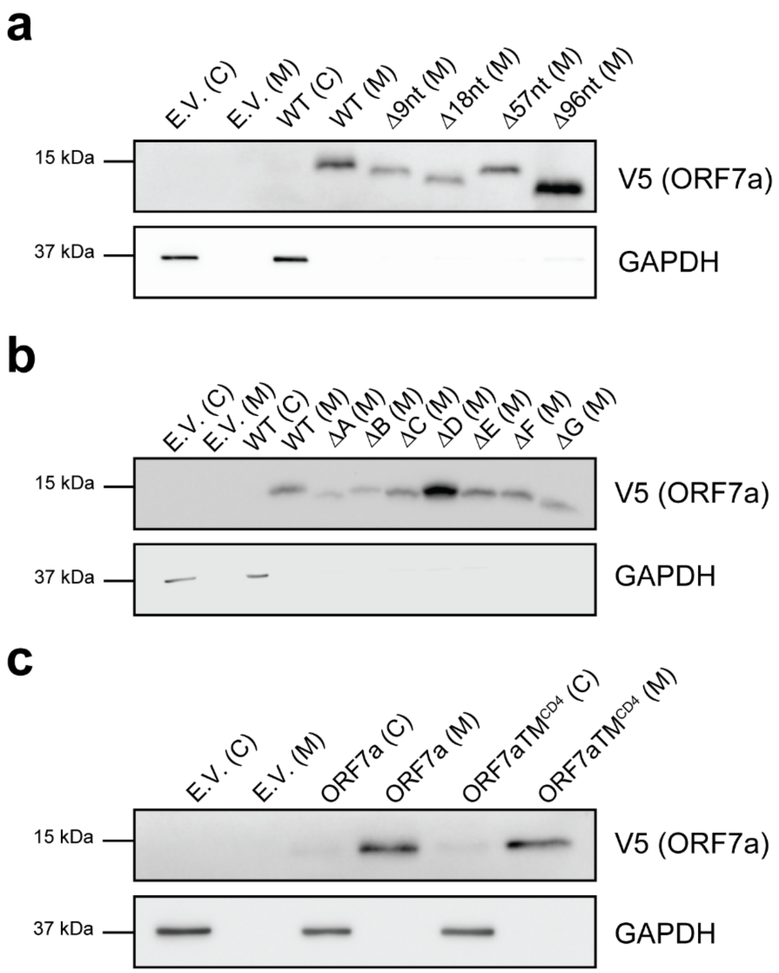

Supplementary Figure 6. **SARS-CoV-2 ORF7a naturally occurring deletion variants and β-sheet deletion mutants localize at cellular membranes.** 293T cells were transfected with the various naturally occurring SARS-CoV-2 ORF7a variants in **a**, SARS-CoV-2 ORF7a β-sheet deletion mutants in **b**, and SARS-CoV-2 ORF7aTM^{CD4} chimera in **c** shown in Fig 8. Cells were harvested and integral membrane proteins were extracted using the MER-PER Plus membrane extraction kit followed by immunoblotting using anti-V5 (SARS-CoV-2 ORF7a detection) and anti-GAPDH (as a marker for the purity of the membrane fractions). In **a** and **b,** both membrane (M) and cytosolic (C) fractions are included for empty vector (E.V.) and wild type SARS-CoV-2 ORF7a (WT) while only membrane fractions are included for SARS-CoV-2 ORF7a variants and mutants. For **a**, **b** and **c**, the representative immunoblot images for n = 2 independent experiments are shown. Uncropped blots are in Source Data. (empty vector, E.V.; wild type SARS-CoV-2 ORF7a, WT; SARS-CoV-2 ORF7aΔ9nt, Δ9nt; SARS-CoV-2 ORF7aΔ18nt, Δ18nt; SARS-CoV-2 ORF7aΔ57nt, Δ57nt; SARS-CoV-2 ORF7aΔ96nt, Δ96nt; SARS-CoV-2 ORF7aΔA, ΔA; SARS-CoV-2 ORF7aΔB, ΔB; SARS-CoV-2 ORF7aΔC, ΔC; SARS-CoV-2 ORF7aΔD, ΔD; SARS-CoV-2 ORF7aΔE, ΔE, SARS-CoV-2 ORF7aΔF, ΔF; SARS-CoV-2 ORF7aΔG, ΔG)

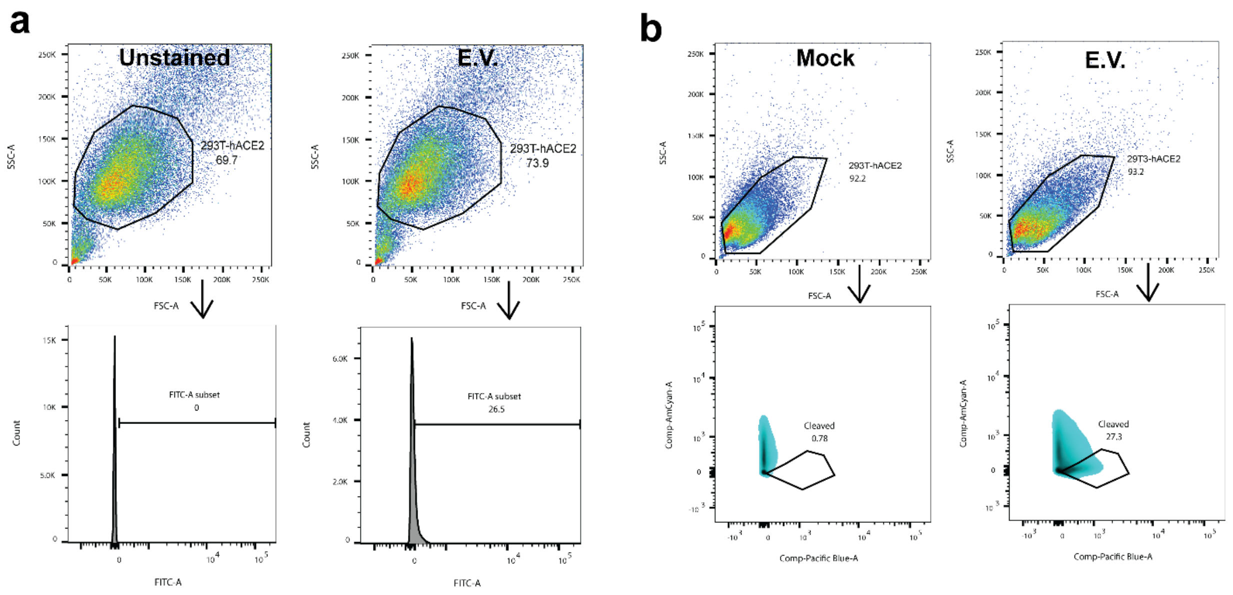

Supplementary Figure 7. **Flow cytometry gating strategy. a** FITC positive single cell population were gated for Virus FITC labelling assay in Fig. 4a. Gate was drawn on unstained sample on the FACS plot. **b** Pacific blue positive (cleaved CCF2) cell populations were gated for BlaM-Vpr fusion assay in Fig. 4c. The fusion gate was drawn on the mock- infected sample.

Supplementary Table 1: Primers used for generating SARS-CoV-2 ORF7a variants

Constructs	Primers used
ORF7aΔ9nt	5'-GTAGCCGCCATAGTCTTTATCAC-3'/ 5'-AATTGGGCTATACAACTCTTGC-3'
ORF7aΔ18nt	5'-GCATGCCCCGATGGTGTTAAACACG-3'/ 5'-AAAACACGTCAAGGCGAACTTGTTG-3'
ORF7aΔ57nt	5'-GTGTCCCCCAAACTGTTCATACGA-3'/ 5'-CTGGGTACTAAAACACGTCA-3'
ORF7aΔ96nt	5'-AAACTGTTCATACGACAAGAGG-3'/ 5'-GTTGTCTGCAAGAGGGTGGA-3'
ORF7aΔA	5'-CGGGGGACAACCGTACTGCT-3'/ 5'-GCATGTAGCCAGGGTTATGAG-3'
ORF7aΔB	5'-CCCTGTAGCAGCGGCACT-3'/ 5'-CCGGACACATTCCTGGTAATG-3'
ORF7aΔC	5'- AATTCCCCCTTCCACCCTCTTGC-3'/ 5'-GCTGCTACAGGGCTCTTTCAG-3'
ORF7aΔD	5'-GACAACAAGTTCGCCTTGACG-3'/ 5'-GAAGGGGGAATTACCTTCATAAGTG-3'
ORF7aΔE	5'-TTTAGTACCCAGTTCGCTTTTGC-3'/ 5'-GTCTGCAAGAGGGTGGAAG-3'
ORF7aΔF	5'-GATGGTGTTAAACACGTGTACC-3'/ 5'-AAAACACGTCAAGGCGAAC-3'
ORF7aΔG	5'-TCTGTGTCCCCCAAACTG-3'/ 5'-ATCGGGGCATGCAAAAGCGAAC-3'
N-V5 SARS CoV-2 ORF7a	V5/His-deletion F 5'-TGAGTTTAAACCCGCTGATCAG-3'<br>V5/His-deletion R 5'-TTCGAACCGCGGGCCCTCTAGAC-3'<br>N-V5-7a-F 5'-GAACTGTATCATTACCAGGAATGTGTCC-3'<br>N-V5-7a-R 5'-CGTAGAATCGAGACCGAGGAGAGGGTTAGGGATAGGCTTACCGCATGTAGCCAGGGTTATGAGG-3'
